# The Sustainability Challenge of Food and Environmental Nanotechnology: Current Status and Imminent Perceptions

**DOI:** 10.3390/ijerph16234848

**Published:** 2019-12-02

**Authors:** Gitishree Das, Jayanta Kumar Patra, Spiros Paramithiotis, Han-Seung Shin

**Affiliations:** 1 Research Institute of Biotechnology & Medical Converged Science, Dongguk University-Seoul, Ilsandong-gu, Gyeonggi-do 10326, Korea; 2 Department of Food Science and Human Nutrition, Agricultural University of Athens, GR-11855 Athens, Greece; 3 Department of Food Science and Biotechnology, Dongguk University-Seoul, Ilsandong-gu, Gyeonggi-do 10326, Korea

**Keywords:** antimicrobials, biosensors, electrospun nanofiber, food processing, nanotechnology, sustainability, environment, human health

## Abstract

Nanotechnology is a connection among various branches of science with potential applications that extend over a variety of scientific disciplines, particularly in the food science and technology fields. For nanomaterial applications in food processing, such as antimicrobials on food contact surfaces along with the improvement of biosensors, electrospun nanofibers are the most intensively studied ones. As in the case of every developing skill, an assessment from a sustainability point of view is necessary to address the balance between its benefits to civilization and the unwanted effects on human health and the environment. The current review aimed to provide an update regarding the sustainability of current nanotechnology applications in food science technology, environment, and public health together with a risk assessment and toxicity evaluation.

## 1. Introduction

Nanotechnology was first hinted at by Richard Feynman during a speech at the annual meeting of the American Physical Society in December 1959. Nanotechnology concerns the use of nanomaterials at a nanometric scale in order to take benefit of the specific physico-chemical properties occurring in this size range. Over the next two decades, the theoretical knowledge and analytical tools for nanotechnology were established, which led to the discovery of fullerenes and carbon nanotubes a few years later. Nanotechnology, being the intersection between physics, chemistry, materials science, engineering, and modern molecular biotechnology, has a number of prospective uses. However, every emerging technology needs to be balanced between the benefits for human civilization and its unwanted effects on environment and life. In the following paper, an attempt was made to present this balance with reference to food nanotechnology by discussing the most typical applications and also discussing advances in green biotechnology together with risk assessments and toxicity evaluations of novel nanomaterials for the purpose of legislation as well as public acceptance in terms of food.

## 2. Nanotechnology and Its Potential Applications in Food Science Technology, Environment, and Human Health

**Figure 1:**
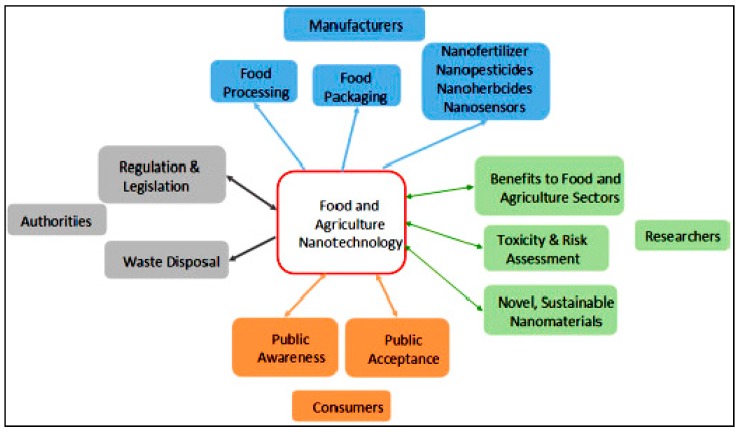
Application of food nanotechnology in various fields. Reproduced with permission from He et al. [5] (originally Figure 1).

Nanotechnology is extensively applied in the everyday life of human beings in almost all fields. It has appeared as a high-tech development in the field of agriculture and food with the potential to increase global food production along with an increase in the nutritional value, quality, and safety of food [1,2,3]. Progress in the arena of nanotechnology has enabled a quite wide and diverse variety of applications in food technology which includes food additives, food safety, nano-delivery systems, biosecurity, nanotoxicity, etc. [1,4,5,6,7]. A number of potential applications for nanotechnology in the food and agriculture sector has been presented by He et al. [5] (Table 1, Figure 1). The use of nanomaterials as processing aids, antimicrobials for surface contact with foodstuffs, and also in the manufacture of biosensors and electrospun nanofibers are the most expansively studied factors which are discussed in the current review along with their pros and cons.

### 2.1. Nanomaterials in the Food Processing Sector

There have been enormous uses of nanotechnology in the food processing sector which include the formulation of novel functional nanomaterials for applications in the food, microscale and nanoscale processing, manufacture of new foodstuffs with enhanced properties and various design of instruments and methods for their possible use in bio-safety and food security [29]. Nanotechnology offers effective approaches in food processing regarding improvement of physico-chemical characteristics of foodstuffs along with improvement in nutrient constancy and bioavailability. Some of the uses of nanotechnology in food sector industries are discussed below. Titanium dioxide and silica dioxide have been exclusively used as processing aids and their use as food additives is allowed within the European Union under the codes E171 and E551, respectively. Titanium dioxide is used mostly as a color enhancer. Weir et al. [30] reported that the highest TiO_2_ content (normalized per serving) was estimated in candies, including chewing gums, chocolate, etc., reaching as higher as 100 mg Ti, whereas dairy products contained less than 0.06 mg Ti. Silicon dioxide, in contrast, is basically used as an anti-caking agent; however, its use in food model preparation for xenobiotic analysis offers significant advantages [31]. Recently, the French government has banned the sale of food products containing TiO_2_ starting from 1 January 2020 [32]. Nanoencapsulation (i.e., encapsulation in a protective envelope of a nanometric scale) may offer significant advantages and new possibilities. The variety of nanocarriers, nanoencapsulation approaches, conditions, and formulae must be nominated as per the food matrix characteristics and the type of encapsulated compound [33]. Research is primarily dedicated to the effective incorporation of lipophilic compounds such as fatty acids, antioxidants, carotenoids, and vitamins [34,35]. A wide range of delivery systems has been described to expand the compatibility between the food matrix and the bioactive compound, provide adequate protection against chemical and physicochemical degradation during specific processing or storage conditions, enhance controlled release upon specific environmental stimuli, and to prolong the antimicrobial potential of the encapsulated antimicrobial compounds. These are the principles on which the structural design of systems for effective delivery of bioactive compounds have been comprehensively studied [36,37].

### 2.2. Nanomaterials in Food Contact Surfaces

The use of nanocomposites for food contact surfaces has become an area of intensive research. More accurately, the antimicrobial potential of metals and their oxides currently employed as well as their consequences on the mechanical and thermal properties on packaging has been studied at length. Regarding the former, the antibacterial potential of nanoscale silver is already known. There are numerous instances of silver incorporation in glass and graphene oxide-inhibiting biofilm formations [38] and in packaging ingredients, like polyvinylpyrrolidone (PVP), cellulose, low-density polyethylene, etc., that exhibit good antibacterial activity against both Gram-positive and Gram-negative species and, in some cases, enhancing the physicochemical stability of the foodstuffs. Another class of compounds, termed photocatalytic nanoparticles, has also been extensively considered. Moreover, the uses of TiO_2_-based polymer coating are effectively applied against biofilm-forming foodborne pathogens [39]. Apart from using metals and their oxides, nano-emulsions have been utilized in improving the antimicrobial potential of compounds. Otoni et al. [40] have described the development of edible films from pectin, papaya puree, and cinnamaldehyde emulsions. Donsi et al. [41] created a film consisting of modified chitosan and containing a nano-emulsion of mandarin essential oil. Besides the antimicrobial activities, nanocomposites are also being utilized to significantly improve basic packaging properties. These properties include protection from physico-chemical or microbiological quality deterioration that is generated from exposure to environmental stimuli. This improvement is accredited to the enhancement of the barrier properties against gases, volatile compounds, and moisture migration along with mechanical and heat resistance [42].

### 2.3. Nanotechnology in Quality and Safety Management of Food

The invention of improved biosensor-based nanotechnology has enabled its potential applications in food-based safety management for detecting both the chemical and biological contaminants. The most extensively studied approaches involve the exploitation of silicon-based nanowires on the basis of their biocompatibility and tunable electrical properties and gold nanoparticles due to the fact of their biocompatibility, ideal optical performance, controlled manufacture, and carbon nanotubes, and their dual application in electrodes and transducer components.

Contaminants, such as allergens, toxins, and pesticides, have been efficiently detected using such biosensors. Regarding allergen detection, improvements have included methods for the discovery of the Ara h1 peanut allergen in chocolate candy bars through a nanobead-enhanced optical fiber surface plasmon resonance biosensor [43], foreign protein contamination (ovalbumin) in whole milk by combining immunomagnetic separation and surface-enhanced Raman scattering [44], and soy protein in soy products by a long-wavelength fluoroimmunoassay by means of a conjugate made up of anti-soy protein antibodies bound to nile blue color doped silica nanoparticles [45].

The enhancement of biosensor technology for bacterial and mycotoxin detection has drawn specific attention [46,47]. Efficient mycotoxin detection was also conveyed by many authors [48,49]. Several biosensors have been developed for pesticide detection also [50].

### 2.4. Electrospun Nanofiber of Food Interest

**Figure 2:**
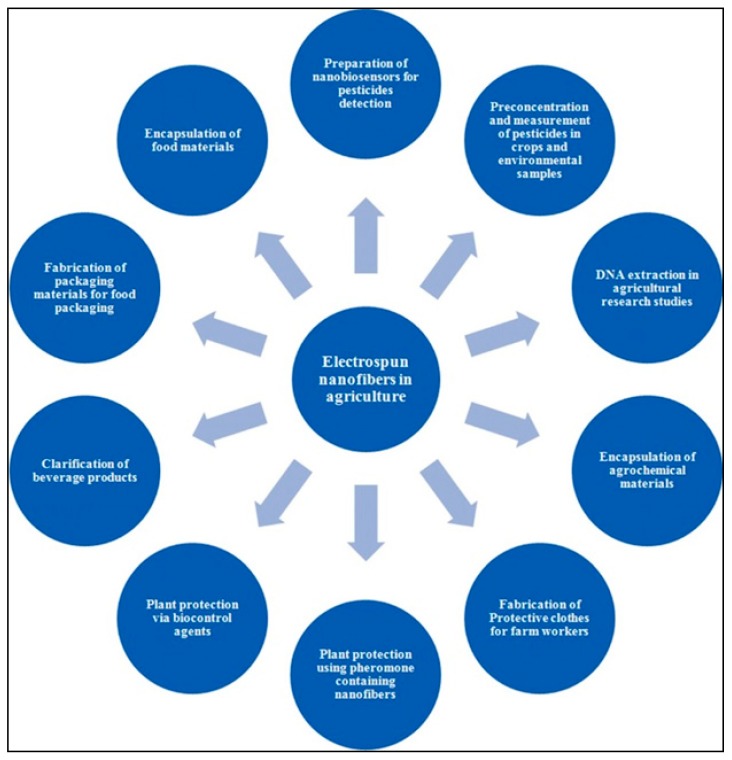
Applications of electrospun nanofibers in agriculture and food. Reproduced with permission from Noruzi 2016 [54] (originally Figure 2).

There is a growing interest in the use of electrospun nanofibers in food sector industries, particularly in the encapsulation of new food ingredients, enzymes, and other types of bioactive compounds along with the electrospinning of biopolymers for food additives, novel packaging, food sensors, food coatings, and flavor enhancement, etc. (Figure 2) [51,52,53,54,55,56].

These applications include active food packaging and preservation of nutrients, enhancing the texture and nature of the food, etc. There are a number of examples of the use of electrospun fibers in the food sectors, such as the addition of antimicrobial agents to electrospun fibers and the use of them in packaging materials for increasing the shelf-life of foods. Natural polymers, such as alginate, chitosan, collagen, gelatin, etc., are being electrospun and tested for their medical applications. Furthermore, food materials, such as zein, soy protein, whey protein, etc., are also being electrospun for their potential applications in the food sector [57,58]. Additionally, intelligent active packaging materials are also being created by electrospun processes for the integration of biosensors into fibers to indicate the expiry date of food products [59]. Fabra et al. [60] reported the use of a bio-based polyester multilayer packaging material with high barrier interlayer electrospun zein nanofibers for food packaging applications. Another application of electrospinning technology is in the case of chocolate making, where the use of electrospinning results in a lower amount of chocolate sauces and the production of fiber particles give varied texture and mouth feeling as compared to bulk chocolate particles [53]. Kriegel et al. [61] introduced eugenol into polyvinyl alcohol and cationic chitosan blended with a Gemini surfactant (Surfynol 465) and tested its promising antibacterial activities [61]. Conservation of active bioactive compounds through a process of encapsulation in the electrospun fibers is one of the most extensively studied fields in the application of electrospun nanofibers in food technology, and it is considered one of the most efficient techniques to protect highly sensitive compounds from various adverse environmental conditions [55]. Folic acid is one example of this application and its beneficial effects, as without any coating it is vulnerable to degradation when exposed to light and acidic conditions. However, when it is encapsulated within sodium alginate-pectinpoly nanofibers, almost 100% of the folic acid is retained after 41 days of storage in the dark at pH 3 as presented by Alborzi [62]. Apart from encapsulation of vitamins and minerals, electrospun fibers techniques are also used for delivery of probiotic bacteria [55,63]. Liu et al. [64] used an aqueous solution containing two edible polysaccharides, pectin and pullulan, for encapsulation of probiotic bacteria *Lactobacillus rhamnosus* GG [64].

### 2.5. Antimicrobial-Rich Nanoparticles in the Food Sector

Spoilage of food materials is caused due to the contamination of food that leads to the growth and proliferation of pathogenic microorganisms, such as bacteria, fungi, food- and water-borne pathogens, etc., which results in the loss of quality of the food [65,66]. Basically, the contamination of food materials is caused due to the fact of exposure to the environment, faulty food processing, and low-quality packaging [65,66]. In order to tackle such issues, there is a need for the development of effective antimicrobial food processing and packaging material which should be safe, effective, and low cost. Besides, the safety evaluations for the active antimicrobial food packaging materials equipped with nanoparticles which can effectively prevent the proliferation of pathogens and protect food from the adverse environment along with increasing the shelf-life of the foods is essential for the future. In such cases, nanomaterials can play a significant role in contending with harmful pathogens and in protecting food [65,67]. Recently, a number of nano-based antimicrobial agents have been tested as food packaging materials, and they have been proven to show enhanced properties such as thermal stability, pH resistance, and other physico-chemical potentials [65,67,68,69,70]. There are several ways of using the antimicrobial compounds as packaging materials in food packaging systems which include the addition of a packet of volatile antimicrobial agents in the packing system which will diffuse slowly into the packet and provide protection to the food from external contaminations. Another way is to directly mix the antimicrobial agents into the polymers used as packaging materials. The other way is to coat the antimicrobial compounds on the surface of the packaging materials or utilize antimicrobial packaging materials directly [66,70,71]. However, effective safety management of these materials is essential in order to protect human health and the environment. Possible mechanisms of action for the effectiveness of antimicrobial agents depends on the controlled release of the active compounds into a system that can provide a durable antimicrobial packaging material, and this can be achieved by the use of nano—micro-structures, such as nanofibers, nano-capsules, and micro-capsules, in the packaging system which helps in the gradual release of the active compounds and also provides mechanical potential for the packaging materials [70]. These types of improved materials are also equipped with smart technologies, such as indicators and dyes, which shows the quality of the product, durability, temperature, pH, and degree of contamination of the food [70,72].

## 3. Sustainability of Food Nanotechnology

**Figure 3:**
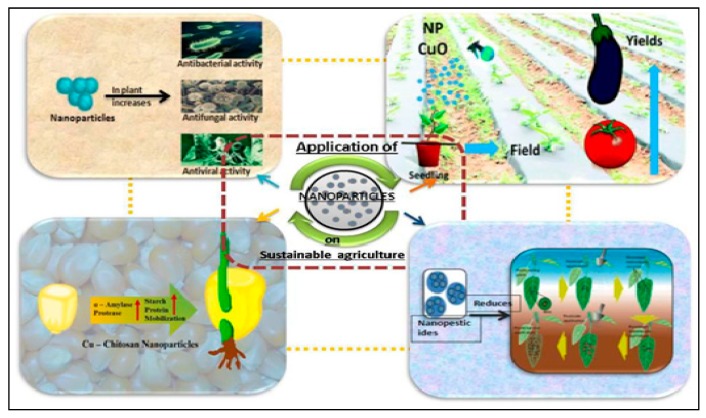
Consequences of nanoparticles in sustainable agriculture. Reproduced from Prasad et al. [74] under the Creative Commons Attribution License (originally Figure 3).

A rapid development in novel nanomaterials and related applications has been witnessed over the last decade. Moreover, this tendency is likely to continue further in the future. A number of promising opportunities have been identified for nano-based technologies which are intended for the improvement of sustainability in agriculture and food systems (Figure 3) [73,74]. These include sensors for testing chemicals, measuring physical, chemical, or biological properties, and for detecting pathogens or toxins in products; advanced techniques for detection and control of harmful pathogens and to increase food safety; technology for water treatment in agricultural fields; nano-based fertilizers, etc. [73,74,75]. However, there are many apprehensions concerning their influence on the environment and human health. Addressing these concerns, the European Food Safety Authority (EFSA) has developed and published a practical approach for real risk assessment on the use of engineered nanomaterials in food and food chain [76]. Within this document, the lack of consistent detection methods, identification, and classification of engineered nanomaterials, especially in multifaceted ecological samples, is mentioned. This issue was also recently talked about by the Organization for Economic Co-operation and Development [77].

The aim of green nanobiotechnology has been adequately presented by Hutchison [78] and Maksimovic and Omanovic-Miklicanin [79]. These may be abridged into two cornerstones: the enlargement of positive effects on the health of humans and well-being and the diminishment of the hostile effects on the environment. Nevertheless, this attitude is significantly hindered by the aforementioned lack of reliable methodology and our inadequate knowledge of the factors which are responsible for the toxic properties of nanomaterials. Study of the structure of various food products at the nanoscale range is a developing area in the field of nanotechnology and, in the near future, it will be a reality to assess the food structure and develop new food materials at the nanoscale range.

### 3.1. Nano-Based Sensors

Nano-based sensors and probes have proved to be beneficial for the improvement of agricultural productivity as well as in food protection and preservation [73,74,80,81,82]. There are numerous examples of nano-based sensors and devices that detect various types of pathogens, toxins, and contaminants in food products and in packaging materials [74,75,82,83]. Regardless of several remarkable achievements, accomplishing the careful and delicate recognition of specific pathogens and toxicants in food remains challenging. The capacity to differentiate between live and dead pathogenic microbes in the food system among a large number of pathogens is always challenging, and it needs to be studied extensively [84]. Furthermore, the manufacture of specific types of nanosensors targeting specific functions in the food system is also challenging [85].

### 3.2. Nano-Based Control of Pathogens

The application of nano-based materials in food packaging, for protection against harmful pathogens and to increase the shelf-life of food materials by nano-coating and smart packaging, is commercially used [86,87,88]. However, there are a number of issues which prevent the smooth implementation of these nano-based materials for food safety. The exact mechanism of such effects on pathogens is not fully understood. Furthermore, the effect of environmental parameters, such as temperature, pH, light, and excretes from food, while packaging is also not properly explained [89]. Finally, the safety of nano-empowered packaging materials which are used straightway on food products and food processing equipment needs to be proved in order to avoid any unintentional negative results on human health [86,89].

### 3.3. Nano-Based Fertilizers

There is a current and growing body of literature on the development of nano-based materials as nano-fertilizers for agrochemical delivery [90,91,92,93]. Notwithstanding being an extremely active research field, approaches to ensuring targeted delivery to specific organisms through the use of biological materials, such as antibiotics and other hormones or materials, to be triggered at extreme environmental conditions are usually lacking. A number of challenges, such as the nature of interactions between plants and nanomaterials, the effect of nanomaterials on plant growth, the nutritional value of the food as well the quality, are still not clear and, thus, prevents the effective use of smart nano-based fertilizers in agriculture [73,92].

### 3.4. Nanoparticle Toxicity

**Figure 4:**
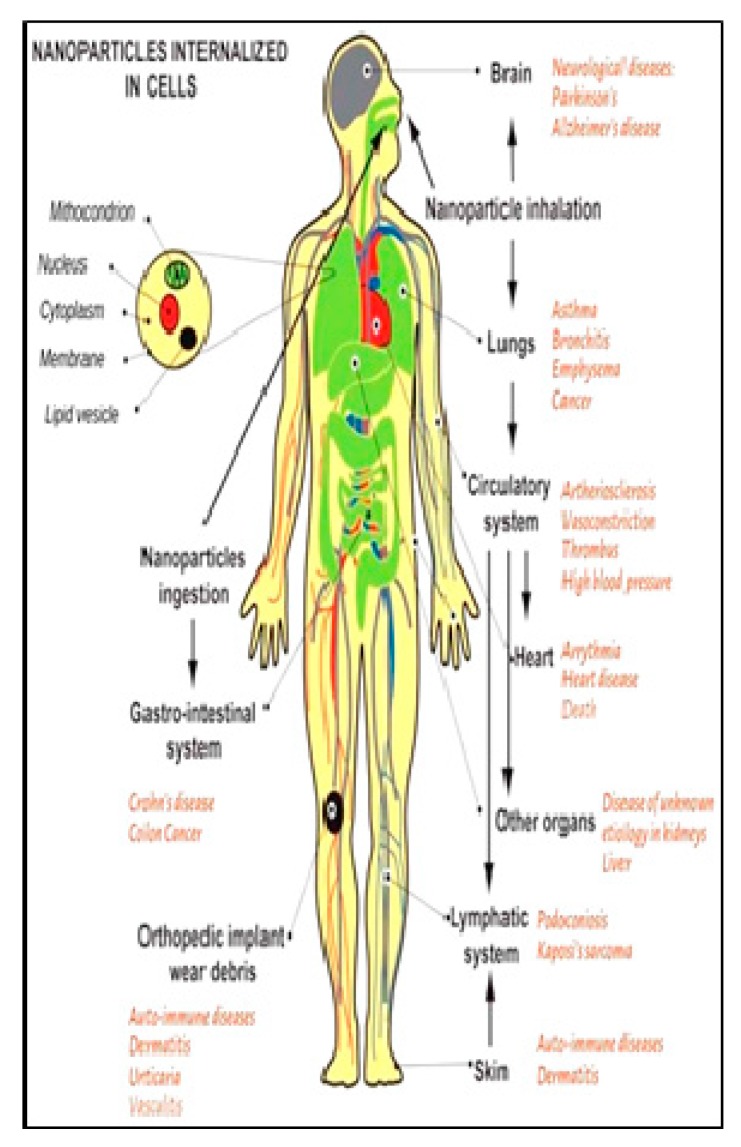
Various types of contact paths linked to nanoparticles and various diseases as proven by epidemiological and clinical studies. Reproduced with permission from Buzea et al. [98] (originally Figure 7).

The cause of nano-toxicity and its future nature has been extensively studied recently. There are numerous entrance points for release of engineered nanomaterials into the environment which includes direct application to an environmental compartment (either intentionally or through unintentional product degradation), wastewater treatment plant effluent, and wastewater treatment plant sludge [94,95]; yet, it is hard to guess the pertinent absorptions of nanoparticles that are released at any given point of time [94]. The amplified nanoparticle utilization in a number of applications including food industries has raised a major concern for food safety and the potential consequences on public health and the environment [96]. The effects on aquatic and terrestrial systems along with associated factors have been recently reviewed by Bundschuh et al. [97]. A number of portions of the human body, especially the skin, lungs, and the intestinal tracts, are in continuous exposure to the outside environment and these parts are vulnerable to nanoparticle exposure [98]. In such cases, the importance of size, shape, chemical composition, solubility, surface properties, and aggregation have been very early recognized [99]. Size-dependent toxicity has also been exhibited in numerous studies involving human lung cells [100]. Nanoparticle shape also significantly affects exerted toxicity [101,102,103]. A variety of nanoparticles, with respect to their size and configuration, could be highly lethal to cells by causing oxidative stress or/and organelle damage [98]. The effect of surface properties on toxicity level has also attracted significant attention, since a variety of coating ingredients, such as proteins, polysaccharides, various surfactants, and citric acid, have been effectively applied [104]. A better understanding of the effect of nanoparticles on various parts of the human body is presented in Figure 4. It is shown that exposure of nanoparticles to various organs may cause specific diseases in that particular organ; for example, when nanoparticles are inhaled, they may cause diseases like emphysema, bronchitis, lung cancer, and neurodegenerative diseases, and further, when intestinal tracts are affected by these nanoparticles, they may result in cancer-related diseases [98,103].

Besides, a number of risk assessment efforts have been undertaken in order to predict the amount of nanoparticles exposed to the environment from various sources, and a summary of this has been presented [94,105,106] which shows that a large dynamic range of nanoparticles are exposed to the environment, and it requires an accurate methodology to measure it. A multidisciplinary tactic which merges experimental, computational, and theoretical methods could be helpful in finding a risk assessment method in order to confirm the eco-toxicological issues linked with the engineered nanoparticles and their exposure to food and the environment.

### 3.5. Operational Approaches

Detection of nanomaterials in the food and various environmental products is a very challenging task, mostly due to the fact of their reactive nature and the concomitant transformations. An effective analysis would include a sample preparation step that would facilitate detection and characterization. Ideally, it should remove any interfering substances and preserve the state and nature of the nanomaterial; however, it is influenced by the analytical step that follows. In general, sample preparation includes a number of steps such as the homogenization of the sample, extraction, and stabilization of the nanomaterials. The type of solvents used in the initial step may affect the second step significantly. Thus, apart from the matrix, the morphological characteristics of the nanomaterial and the subsequent separation technique need to be considered. In Table 2, representative studies for the detection of inorganic and organic nanoparticles in the biological samples are summarized. In the case of inorganic nanoparticles, matrix interference is removed by chemical or enzymatic digestion that may be assisted by microwaves or sonication followed by solid or liquid phase extraction process. After extraction has taken place, a fractionation method precedes and is united with a detection one.

Detection was mostly achieved through microscopic and spectrometric techniques. Regarding the microscopic techniques, electron microscopy may provide information on the morphological characteristics and nature of the nanomaterials. However, sample preparation introduces high uncertainty [117]. This issue was addressed, at best, in the liquid samples by application of environmental (or atmospheric) scanning electron microscopy which can be carried out with a basic sample preparation method. Light scattering was initially regarded as a harmonizing approach. However, unequal distribution of the size of the nanoparticles hinders the potential in the complicated samples. This limitation was only partially addressed, at least in liquid samples, by nanoparticle tracking analysis (NTA) [118]. As far as spectrometric techniques are concerned, they may be provided with detection, identification, and quantification of nanoparticles. Inductively coupled plasma-mass spectroscopy (ICP-MS), single-particle ICP-MS (spICP-MS) as well as matrix-assisted laser desorption/ionization-time of flight (MALDI-TOF) are mostly used due to the satisfactory size and concentration limit of detection [119] but no information regarding size, shape or aggregation may be obtained. However, this limitation is overruled by coupling with a fractionation technique [120].

Specific characterization of organic nanoparticles, such as fullerenes and carbon nanotubes, have been comprehensively assessed; however, graphene and graphene oxides have not been so exclusively studied. In the latter case, only limited literature is currently available. The most effective approach was described by Doudrick et al. [115] which included chemical reduction of graphene oxide, allowing a competent extraction and separation from background carbon and reliable quantification by programmed thermal analysis (PTA). Information on the size, shape, and degree of accumulation of organic nanoparticles is acquired as in the case of inorganic ones through electron microscopy, light scattering, and NTA techniques. Regarding fullerenes, the separation and extraction process together with the detection and quantification approaches were appraised by Astefanei et al. [121]. In brief, in complex matrices, removal of proteins and surfactants precedes liquid–liquid extraction that is usually employed as such or in combination with ultrasounds using oxidizing agents or salts and toluene. Liquid chromatography coupled with mass spectrometry (LC-MS) is the detection step that is mostly employed. A widespread extraction, separation, and detection process has been described for effective detection and quantification of carbon nanotubes and comprehensively reviewed [122]. Depending on the matrix, a pre-treatment involving chemical or enzymatic digestion, either sonication-assisted or not, is essential. Then, extraction and separation through a variety of approaches, such as asymmetric flow field-flow fractionation (AF4), chromatographic or electrophoretic techniques may take place followed by quantification strategies such as spICP-MS, thermal gravimetric analysis-mass spectrometry (TGA-MS), etc.

Finally, a major challenge is to distinguish between anthropogenic contamination and naturally occurring nanoparticles. In the case of inorganic ones, this may be achieved through the calculation of specific ratios such as Ti to Fe and Ce to La [123,124].

### 3.6. Measurement Issues

The capacity to quantify the presence of nanomaterials in the food system at a particular time period is a very critical issue for its potential application in the food system [125,126]. These quantifications of the nanomaterials comprise both the preparation and storage of the food products, along with its digestion and channel through the alimentary canal of the digestive system of the humans which is itself a various complicated issue due to the multifaceted nature of the human body and the thermodynamic instability of the nanomaterials [125]. It is also not clear what factors in specific are needed to be quantified. A number of factors are accountable for the establishment of a highly applicable method in the measurement of nanomaterials in the food system that comprise the case of how nanomaterials are added, and it is always essential to identify them whether they are natural or added from external sources as food additives, enhancers or emulsions. Besides, the multifaceted nature of the human alimentary canal also creates further concerns in the measurement and classification of the nanomaterials [125]. A number of analytical methods particularly in a combined form are applied to basically measure the nanomaterials in the food [125,127]. These methods include microscopy (transmission electron microscopy, scanning electron microscopy), chromatography, spectroscopy (X-ray powder diffraction spectroscopy, energy-dispersive X-ray spectroscopy), centrifugation, chromatography, and other related methodologies [128].

## 4. Conclusions

There is numerous evidence for the involvement of the science of nanotechnology in almost all steps of the food chain. In addition, novel nanomaterials along with their applications are expected to emerge within the upcoming years. Consequently, it is imperative to discuss these advancements through a green biotechnology perspective. The essential first step towards this direction is the improvement of the analytical tools that will allow accurate and reliable quantification of the planned nanomaterials in a multifaceted environmental sample. Then only will our understanding regarding their conversions and bio-kinetics be improved, allowing for the design of safer nanomaterials with reduced environmental impact.

## Figures and Tables

**Table 1:** Different examples of nano-based food products.

Sector	Application	Nanomaterials	Manufacturer	Current Status	Note	Reference
Food processing	Color additives	TiO_2_		Exempt from certification	<1% by weight of the food	[8]
		Synthetic iron oxide		Exempt from certification	<0.25% (for dogs and cats) and 0.1 (for human) % by weight of the finished food	[8,9]
	Additive or polymer production aid	ZnO, iron oxide, aluminum oxide, silicon dioxide, cobalt oxide, manganese oxide (E530)		Authorized by EC 10/2011	Authorization based on conventional particle size	[10]
		Titanium nitride			No migration reported. Only to be used in PET bottles up to 20 mg/kg	
		Carbon black		Authorized by EC 10/2011; no longer authorized by the US FDA as additives	<2.5% *w*/*w* in the polymer	
	Preservatives	Silver-silica	Nanox Intelligent Materials	FCS Inventory ^a^	FCN No. 1235. <4 ppm by weight of silver as an antimicrobial agent blended into polymers	[11]
	Flavor carrier	Silicon dioxide (E551 ^d^)		Authorized by EC1334/2008	<10,000 mg/kg, excluding foods for infants and young children	[12]
	Marking fruit and vegetables	Silicon dioxide (E551)		Exempt from certification	<2% of the ink solids	[8]
	Anticaking agents	Silicon dioxide (E551)		REG ^b^	<2% by weight of the food	[13]
	Nutritional dietary supplement	Copper oxide, iron oxide			Approved for animal feed	[14]
		ZnO		GRAS ^c^		
Food contact packaging	Pesticides detection	Zinc Oxide QDs		R&D		[15]
	Pathogens detection	Magnetic nano-sensors		R&D		[16,17]
		Plasmonic nano-sensors				[18]
		Fluorescent nano-sensors				[19]
	Toxins detection	Fluorescent nano-sensors		R&D		[20]
		Plasmonic nano-sensors				[21]
		Phosphorescent QDs				[22]
	Edible film/coating	Chitosan/nano-silica coating			Tested on longan fruit	[23]
		Poly-ε-caprolactone			Tested on fresh-cut “Red Delicious” apples	[24]
		Nano-emulsion/quinoa protein/ chitosan			Tested on fresh strawberries	[25]
		Bio-nano-hybrid pectins and LDH-salicylate			Tested on fresh apricots	[26]
		Nano-emulsion with lemongrass essential oil		R&D	Tested on fresh-cut Fuji apples	[27]
		Bentonite (Al_2_O_3_4SiO_2_nH_2_O)		GRAS	US FDA 21CFR184.1155	[28]
	Flame retardation additives, gas barrier, etc. Prevent abrasive wear	Montmorillonite	PolyOne Corporation Nanocor^®^ Inc.	FCS Inventory	FCN No. 1163	[11]
		Montmorillonite chromium (III) oxide	Toyo Seikan Kaisha Limited and Nanocor Incorporated		FCN No. 932	[26]
		Nano-emulsion with lemongrass essential oil	Oerlikon Balzers Coating AG, Oerlikon Surface Solutions AG		FCN No. 1839. For use at a thickness not to exceed 200 nm, not for use in contact with infant formula and human milk	[27]
	Prevent abrasive wear Heating enhancer in polyethylene terephthalate (PET) polymers	Titanium aluminum nitride	Balzers Aktiengesellschaft	GRAS	FCN No. 302. The maximum thickness of the surface coating shall not exceed 5 mm	[28]
		Tin antimony oxide	Nyacol Nano Technologies, Inc.	FCS Inventory	FCN No. 1437. <0.05% by weight of the polymer	[11]

^a^ FCS: Effective Food Contact Substance (FCS) Notifications; ^b^ REG: Food additives for which a petition has been filed and a regulation issued; ^c^ GRAS: Generally Recognized as Safe; ^d^ E numbers are codes of specific substances used as food additives approved by the European Food Safety Authority (EFSA). EC: European Commission; FDA: United States Food and Drug Administration; R & D: Research & Development; Layered double hydroxide. Reproduced with permission from He et al. [5].

**Table 2:** Representative studies for the recognition of nanomaterials in biological entities.

Target Nanoparticle (NP)	Matrix	Sample Preparation	Detection/Quantification Method	Comments	Reference
Ag NPs	chicken meat	sonication followed by proteinase K treatment	SP-ICP-MS	The established method exhibited good performance with respect to trueness, repeatability, reproducibility, and ability to determine Ag NPs transformed into silver sulfide.	[107]
Ag NPs	sock fabric	HNO_3_/H_2_O_2_ digestion	ICP-OES	The sock manufacturing process may control silver release; high silver concentration will end with the wastewater treatment facility limiting the disposal of the biosolids as agricultural fertilizers.	[108]
Cu NPs	topsoil	colloidal soil suspensions digested by HNO_3_/HCl/H_2_O_2_ and microwaves	ICP-MS	The significance of dwell time, background removal, and sample dilution as methods for optimization and recovery maximization were highlighted.	[109]
TiO_2_	water suspended particulate matter	filtration	SP-ICP-MS	TiO_2_ NPs from sunscreens are possibly released into the water but settle into the sediment.	[110]
TiO_2_ NPs, Ag NPs, Au NPs	water	none	SP-ICP-MS	Lime softening followed by alum coagulation collected with powdered activated carbon adsorption resulted in removal of Au and Ag NPs and almost complete of TiO_2_ NPs in wastewater.	[111]
Various fullerenes	wastewater	filtration followed by sonication-assisted toluene extraction and partial evaporation	LC-QqLIT-MS	The established method was characterized as very effective.	[112]
C60 and C70 fullerenes	soil and sediment	sonication-assisted toluene extraction and partial evaporation	UHPLC-HRMS	A fast and sensitive method suitable for the analysis of very complex matrices.	[113]
Various fullerenes	water and sediment	LLE with toluene (water samples); ultrasound extraction and PLE (sediment samples)	UHPLC-MS/MS	An effective approach for fullerene analysis in biological entities.	[114]
Graphene and graphene oxide	wastewater biomass	solubilization followed by thermal digestion and reduction	PTA	The proposed approach provided had promising results.	[115]
SWCN	sediment	sonication in the presence of surfactants	NIRF Spectroscopy	The applicability of this tactic was exhibited.	[116]

NPs: NanoParticles; SP-ICP-MS: single particle inductively coupled plasma mass spectrometry; OES: Optical Emission Spectroscopy; LC-QqLIT-MS: liquid chromatography coupled to a hybrid triple quadrupole linear ion trap mass spectrometry; UHPLC-HRMS: Ultra High Performance Liquid Chromatography coupled with High Resolution Mass Spectrometry; LLE: liquid-liquid extraction; PSE: pressurized solvent extraction; PTA: Programmed Thermal Analysis; SWCN: Single-Walled Carbon Nanotubes; NIRF: Near InfraRed Fluorescence.
